# Clinical observation of laparoscopic surgery for pediatric periappendiceal abscess

**DOI:** 10.3389/fsurg.2026.1886344

**Published:** 2026-10-14

**Authors:** Danjing Sun, Xuechao An, Xiangming Wu, Lili Xiao, Hailong Zhang, Lin Yang, Wenfeng Gao, Jianguo Zhang

**Affiliations:** 1 Department of Pediatric Surgery, Inner Mongolia Autonomous Region Maternal and Child Health Hospital, Hohhot, Inner Mongolia, China; 2 Baotou Medical College, Baotou, China,

**Keywords:** children, clinical observation, IL-6/CRP/PCT, laparoscopic, periappendiceal abscess

## Abstract

**Objective:**

This study aimed to investigate the changes in inflammatory markers such as CRP, PCT, and IL-6 in the treatment of pediatric periappendiceal abscess and to explore more effective treatment strategies by observing the changes in inflammatory and general indicators after laparoscopic surgery and conservative treatment. The findings are expected to provide more precise guidance for clinicians in making diagnostic and treatment decisions.

**Methods:**

This study conducted a single-center retrospective analysis of 80 eligible pediatric patients with periappendiceal abscess treated in our hospital's Pediatric Surgery Department from January 1, 2019, to December 31, 2024. All patients were definitively diagnosed with periappendiceal abscess based on symptoms/signs, serological examinations, and imaging studies (right lower abdominal color Doppler ultrasound, lower abdominal CT), meeting the inclusion criteria. All patients presented with fever ± nausea/vomiting, right lower abdominal pain, rebound tenderness (+), McBurney's point tenderness (+), positive colonic inflation test, and ultrasound-confirmed inflammatory mass in the right lower quadrant. Patients were allocated to surgical treatment group or conservative treatment group according to intervention methods. Clinical efficacy, inflammatory indicators, and general condition changes were compared between groups.

**Results:**

In terms of clinical efficacy, there was a statistically significant difference between children undergoing laparoscopic surgery and those receiving conservative treatment (*P* < 0.05); in terms of inflammatory indicators, both the surgical treatment group and the conservative treatment group showed rapidly declining trends in inflammatory markers including WBC, IL-6, CRP, and PCT (*P* < 0.05), with no significant difference in the rate of decline between the surgical and conservative groups (*P* > 0.05); in terms of general conditions, there was no statistically significant difference in hospitalization days between the two groups (*P* > 0.05), while the time to first flatus and duration of abdominal pain were significantly better in the surgical treatment group compared to the conservative treatment group (*P* < 0.05).

**Conclusion:**

This study demonstrates that laparoscopic appendectomy in children with periappendiceal abscess yields superior outcomes in general recovery compared to conservative treatment (*P* < 0.05), while both groups exhibit equivalent efficacy in inflammatory marker resolution, with rapid declining trends observed post-intervention. These findings confirm that conservative management requires prolonged duration and achieves suboptimal therapeutic efficacy for pediatric periappendiceal abscess; whereas laparoscopic surgery more effectively enhances patients’ immune function, accelerates inflammation resolution, improves treatment outcomes, and significantly shortens clinical management duration, thereby providing precise guidance for clinical decision-making. However, as a single-center retrospective study with limited sample size, further validation through large-scale prospective studies is warranted.

## 1 Introduction

Acute appendicitis is a common acute abdomen in pediatric surgery (1). Pathologically, its pathogenesis is closely related to appendiceal lumen obstruction, local ischemic necrosis, reactive hyperplasia of the appendiceal wall, and gastrointestinal dysfunction. Studies have shown that the incidence of this disease varies regionally, and the severity of the condition, clinical manifestations, and surgical management in children differ significantly due to regional economic disparities (2). Acute appendicitis in children is showing an increasing incidence trend globally, with epidemiological data indicating it accounts for 10%–15% of pediatric acute abdomen cases. Due to children's often unclear or atypical descriptions of abdominal symptoms, delayed detection and diagnosis occur in some cases (3, 4), leading to approximately 4%–10% of children presenting with periappendiceal abscess formation at the time of medical consultation. Previous studies have suggested that its occurrence is associated with factors such as fecalith obstruction in the appendiceal lumen, elevated appendiceal wall pressure due to excessive lymphoid follicle hyperplasia, bacterial invasion, and abnormal local immune responses. Although surgery remains the curative approach, treatment strategies for the abscess stage remain contentious: conservative management advocates anti-infective therapy, drainage, and nutritional support, while laparoscopic surgery emphasizes early eradication of the infectious focus. The controversy centers on the trade-offs in surgical timing selection, postoperative infection risks, and long-term complications. Consequently, the optimal management approach for periappendiceal abscesses in children remains debated (5, 6), with no universally accepted protocols for surgical indications or operative techniques (7). A perspective suggests that surgical intervention for periappendiceal abscess may disrupt the body's inherent immune barrier, potentially exacerbating inflammatory dissemination (8). In some pediatric cases, abscess-induced local inflammatory edema and obscured anatomy can lead to intestinal injury, adhesive bowel obstruction, or even enteric fistula during forced appendectomy (9). Conversely, others argue that conservative management carries risks of intraperitoneal infection dissemination, significantly increasing the likelihood of diffuse peritonitis. Additionally, conservative approaches exhibit slow efficacy, prolonged hospitalization, and an 8%–25% recurrence rate of appendicitis post-recovery (10). A meta-analysis by George et al. revealed that pediatric patients prioritizing conservative management exhibited a lower incidence of postoperative complications than those undergoing immediate surgery after diagnosis. However, the surgically prioritized cohort demonstrated accelerated recovery and shortened hospitalization (11). These findings were corroborated by Blakely et al.'s research (12). Currently, laparoscopic appendectomy or exploratory laparoscopy has become the standard surgical approach for pediatric periappendiceal abscesses, offering superior visualization, minimized incisions, reduced surgical site infection rates, and expedited postoperative recovery (13, 14).

Since 2019, our institution has adopted laparoscopic surgery for pediatric periappendiceal abscesses, accumulating substantial clinical experience with clinically satisfactory outcomes. In contemporary surgical diagnosis of periappendiceal abscesses, hospitals routinely monitor inflammatory biomarkers (15, 16). While white blood cell count (WBC) and neutrophil percentage serve as conventional indicators for appendicitis assessment, there is a pressing need for more precise inflammatory markers to evaluate infection severity in pediatric patients (17, 18). Advances in laboratory diagnostics have demonstrated that biomarkers including C-reactive protein (CRP), procalcitonin (PCT), and interleukin-6 (IL-6) significantly enhance the diagnosis of acute abdominal conditions5. This study therefore selects WBC, CRP, PCT, and IL-6 as primary inflammatory indicators for observation.

To delineate the differential changes in inflammatory biomarkers and prognostic outcomes in pediatric patients with periappendiceal abscesses following conservative management vs. surgical intervention, this study compares the clinical efficacy of both approaches to identify therapeutic strategies that minimize patient discomfort and accelerate recovery. This study will compare changes in inflammatory indicators and general clinical parameters among pediatric patients with periappendiceal abscesses following conservative management vs. surgical intervention, aiming to investigate fluctuations in inflammatory markers (IL-6, CRP, PCT) during treatment and observe the therapeutic efficacy of both approaches.

## 2 Materials and methods

### 2.1 General clinical data

We enrolled 80 pediatric patients with periappendiceal abscesses treated in our pediatric surgery department between January 2019 and December 2024. All patients were definitively diagnosed based on symptoms, serological tests, and imaging studies (right lower quadrant color Doppler ultrasound, lower abdominal CT) and met the inclusion criteria. Clinical manifestations included fever (with or without nausea/vomiting), right lower quadrant pain, rebound tenderness (+), McBurney's point tenderness (+), and positive Rovsing's sign. Ultrasound confirmed inflammatory masses in the right lower quadrant (19). After excluding patients with drug allergies, congenital diseases, or inability to cooperate with treatment, subjects were divided into conservative management (*n* = 35) and laparoscopic surgery groups (*n* = 45). The cohort comprised 46 boys and 34 girls aged 2–11 years (mean age: 5.5 ± 2.8 years), with disease duration of 2–6 days (mean: 3.7 ± 1.2 days). No statistically significant differences existed in baseline characteristics between groups (*P* > 0.05), ensuring comparability.

### 2.2 Inclusion and exclusion criteria

#### 2.2.1 Inclusion criteria

1. Confirmed diagnosis of periappendiceal abscess;
2. Age 2–18 years with no severe cardiopulmonary or renal dysfunction;
3. All treatments (conservative or surgical) administered within the pediatric surgery
4. Department;
5. Complete assessment of inflammatory markers and therapeutic efficacy.

#### 2.2.2 Exclusion criteria

1. Pediatric patients with periappendiceal abscesses receiving incomplete treatment course;
2. Those uncooperative with comprehensive inflammatory marker assessment or
3. Therapeutic efficacy evaluation;
4. Patients lacking definitive diagnosis of periappendiceal abscess.

### 2.3 Therapeutic methods

1. Conservative Treatment Group: Intravenous administration of cefoperazone/sulbactam sodium (100 mg/kg every 8 h) combined with metronidazole in sodium chloride injection (7.5 mg/kg every 8 h).
2. Surgical Treatment Group: Laparoscopic appendectomy performed under general anesthesia in the operating suite post-diagnosis.

### 2.4 Observation indicators

1. Clinical efficacy: After 7 days of treatment, clinical efficacy in children was evaluated based on ultrasound results and symptoms/signs. Clinical efficacy was divided into the following three categories: markedly effective: symptoms/signs completely disappeared, ultrasound showing >70% reduction in abscess size; improvement: symptoms/signs basically disappeared, ultrasound showing 50%–70% reduction in abscess size; ineffective: failed to meet effective criteria.
  Inflammatory indicators: Observed and compared changes and differences in white blood cells, C-reactive protein, procalcitonin, and interleukin-6 levels in children before treatment and on days 3 and 6 after treatment.
2. General conditions: Observed and compared general conditions including hospitalization days, time to first flatus, duration of abdominal pain (from disease onset to post-treatment relief), and compared whether there were statistical differences between the two groups.

### 2.5 Statistical analysis

Statistical analysis was performed using SPSS 22.0 software. Normality tests were conducted for measurement data in clinicopathological parameters. Data conforming to normal distribution were expressed as mean ± standard deviation (), with between-group comparisons using independent samples *t*-test; categorical data were expressed as number and percentage (), with between-group comparisons using *χ*^2^ test or Fisher's exact test. A result of *p* > 0.05 indicated no statistically significant difference between groups, while *p* < 0.05 indicated statistically significant difference. All statistical tests were two-tailed, and *p* < 0.05 was considered statistically significant.

## 3 Results

### 3.1 Baseline data and between-group comparability analysis

A total of 80 children diagnosed with periappendiceal abscess were included in this study, comprising 35 in the conservative treatment group (15 males, 20 females) and 45 in the surgical treatment group (31 males, 14 females). Analysis of baseline characteristics revealed a statistically significant difference in gender distribution (*χ*^2^ = 5.46, *P* = 0.019), while all other variables exhibited overall comparability between the groups.

### 3.2 Analysis of clinical efficacy differences between groups

A total of 80 children diagnosed with periappendiceal abscess were included in this study, with 35 receiving conservative treatment and 45 undergoing laparoscopic surgical treatment. Statistical analysis using SPSS 22.0 showed a significant difference in clinical efficacy between the conservative and surgical groups (*p* < 0.05) by *χ*^2^ test (Table 1), indicating superior efficacy of surgical intervention for pediatric periappendiceal abscess compared to conservative treatment.

**Table 1:** Relationship between clinical efficacy and treatment methods.

Clinical Efficacy	Children with Periappendiceal Abscess (Cases)			*χ* ^2^	*p*
	Total (*n* = 80)	Conservative Group (*n* = 35)	Surgical Group (*n* = 45)		
Markedly effective	53	20	39	8.864	0.003
Imoroved	27	15	6		
No improvement	0	0	0		

p: Significance of χ^2^ test between the training set and validation set.

### 3.3 Analysis of dynamic changes in inflammatory markers and intergroup differences

This study included 80 pediatric patients diagnosed with periappendiceal abscess, among whom 35 received conservative treatment and 45 underwent laparoscopic surgery. Repeated measures ANOVA using SPSS 22.0 on inflammatory markers (WBC, CRP, PCT, IL-6) at preoperative, postoperative day 3, and postoperative day 6 showed:

1. Significant main effect of time: Multivariate tests indicated that all inflammatory marker levels decreased significantly over time (Pillai's Trace: F = 26.521, *P* < 0.001; Wilks’ Lambda: F = 45.581, *P* < 0.001), suggesting a rapid decline in inflammatory response postoperatively regardless of treatment approach.
2. No interaction effect between treatment and time: The treatment-time interaction was not statistically significant (Pillai's Trace: F = 0.691, *P* = 0.700; Wilks’ Lambda: F = 0.692, *P* = 0.699), indicating no significant difference in the rate of decline in inflammatory markers between the surgical and conservative groups (Figures 1–4).

**Figure 1:**
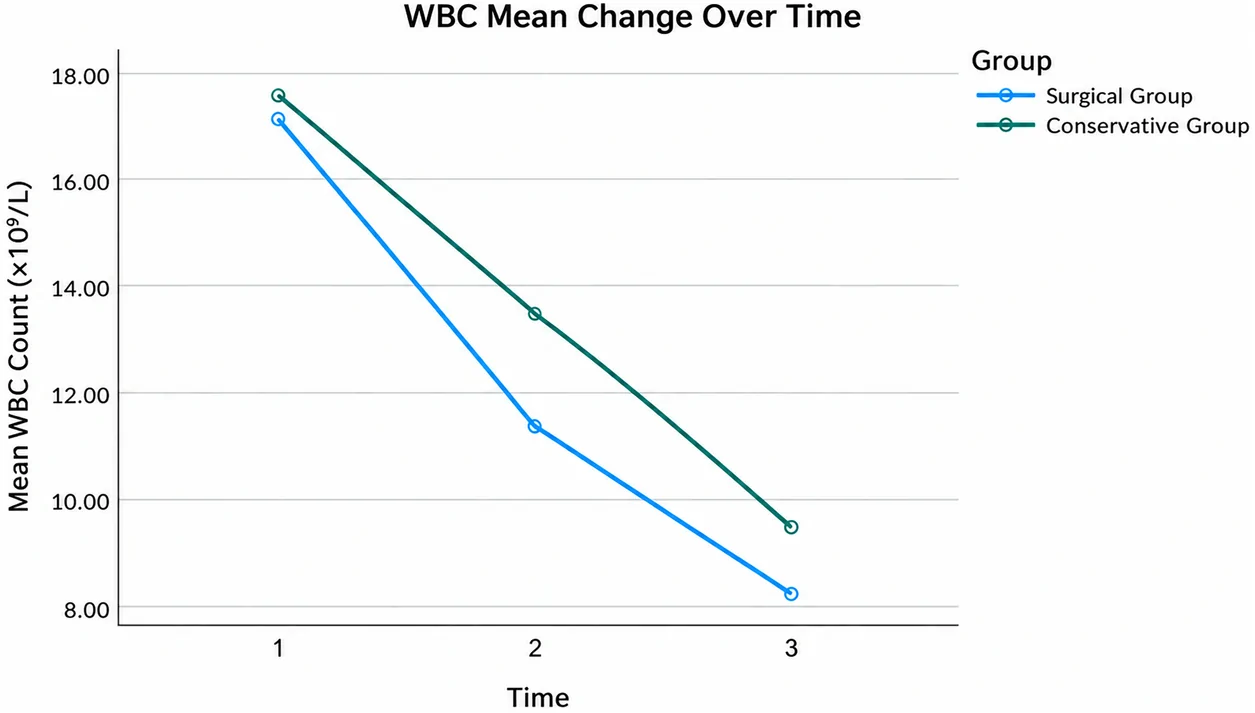
Comparison line chart of WBC changes between groups.

**Figure 2:**
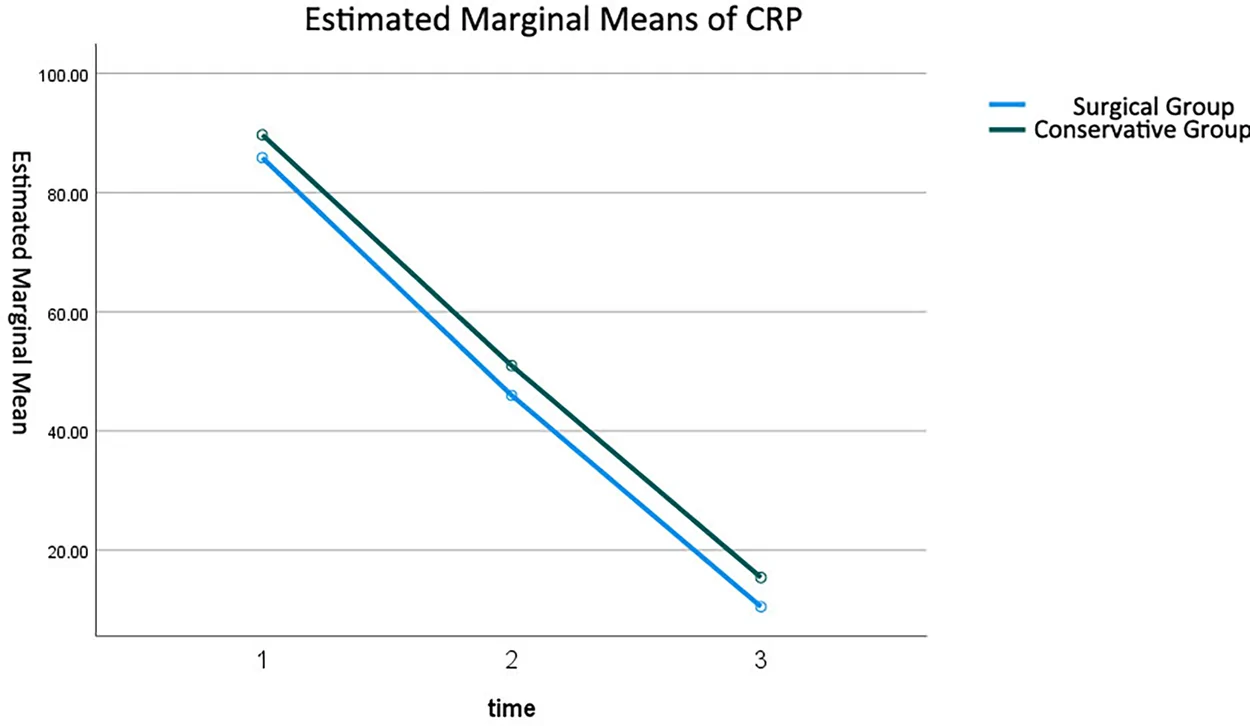
Line chart comparing CRP changes between groups.

**Figure 3:**
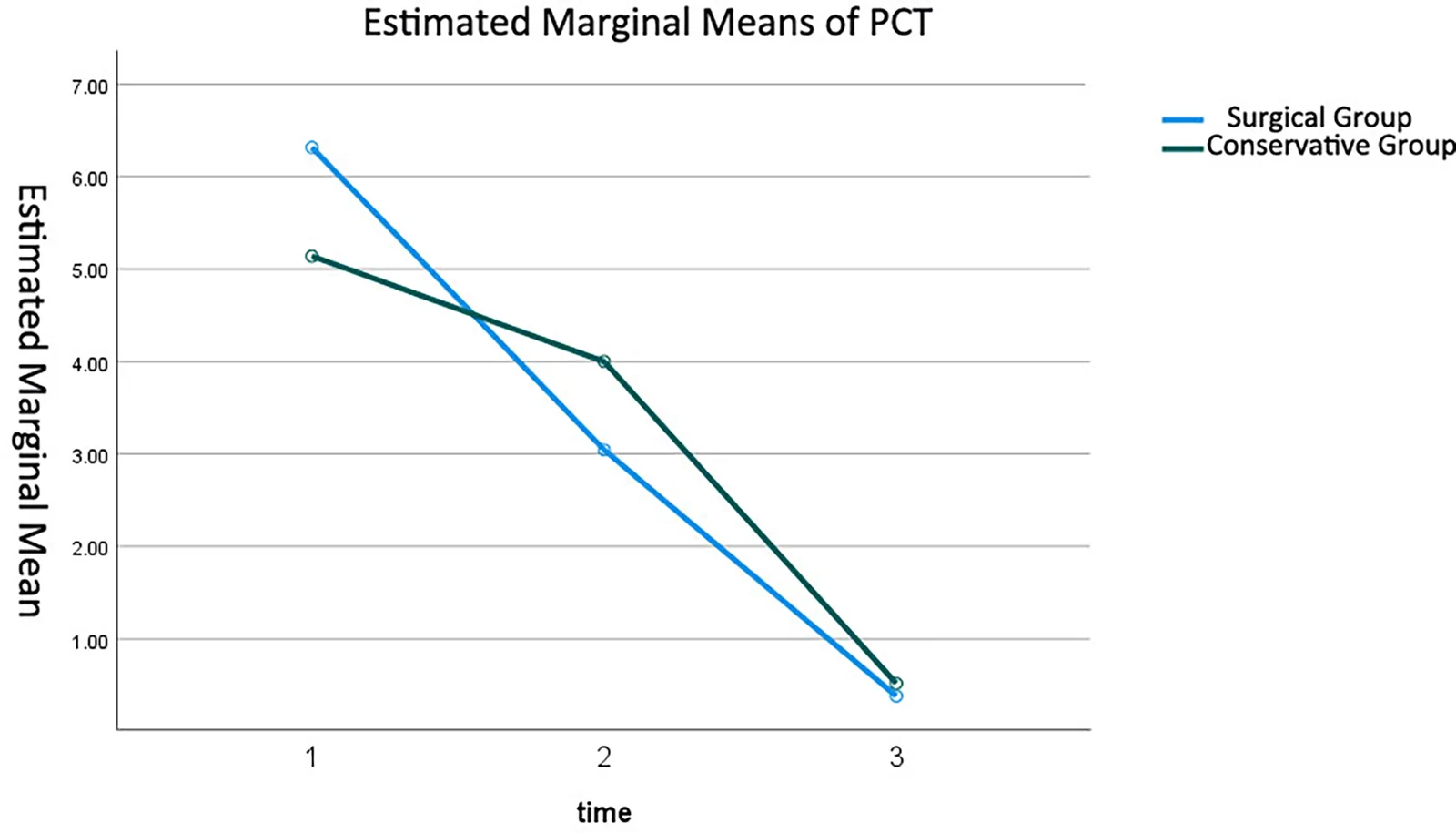
Line chart comparing PCT changes between groups.

**Figure 4:**
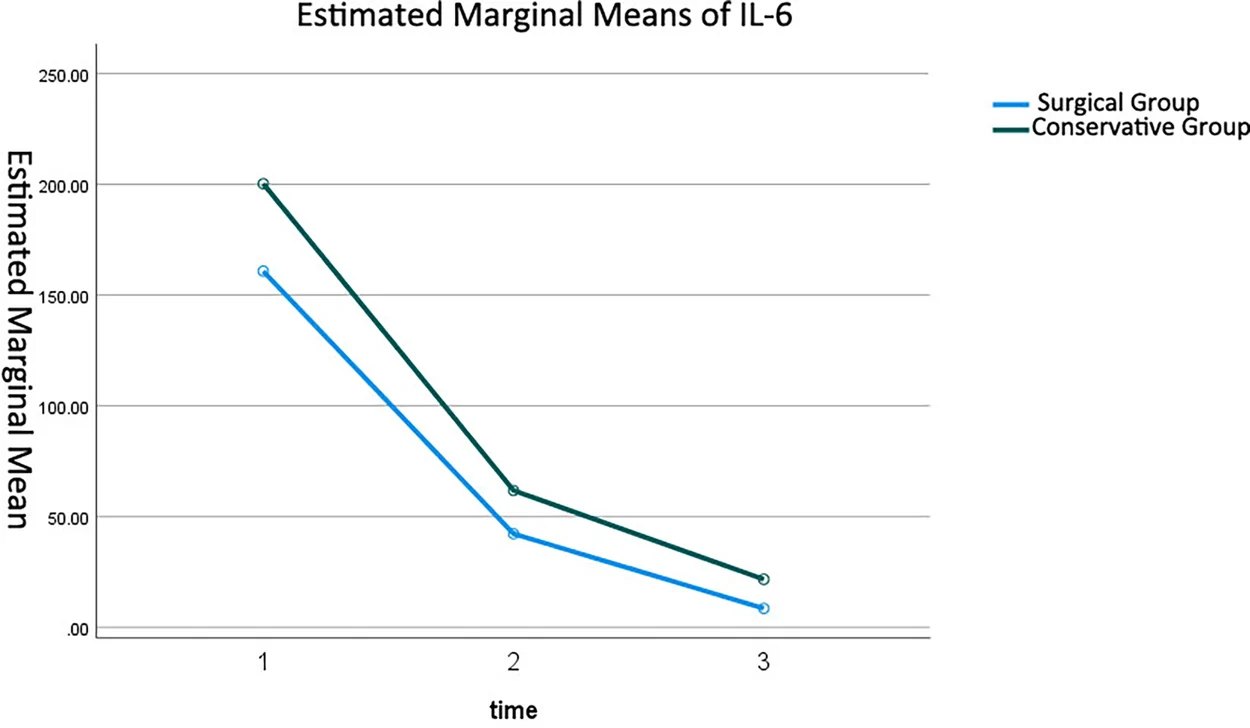
Line chart comparing IL-6 changes between groups.

We further performed t-tests on samples from postoperative days 3 and 6, with the results showing: on day 3, there were no statistically significant differences in WBC, CRP, PCT, or IL-6 between the two groups (all *P* > 0.05); on day 6, there were no statistically significant differences in WBC, CRP, PCT, or IL-6 between the two groups (all *P* > 0.05) (Table 2).

**Table 2:** Effect of treatment methods on inflammatory indicators.

Time point and parameters	Within-group statistics of children with periappendiceal abscess				t	*p*
	Groups	Cases, *n*	Mean	SD		
POD3 WBC	Surgical Group	45	11.42	4.61	−1.960	0.054
	Conservative Group	35	13.49	4.82		
POD3 CRP	Surgical Group	45	45.97	40.52	0.565	0.574
	Conservative Group	35	50.96	37.45		
POD3 PCT	Surgical Group	45	3.04	6.29	0.602	0.549
	Conservative Group	35	3.99	7.91		
POD3 IL-6	Surgical Group	45	42.28	46.65	−1.545	0.126
	Conservative Group	35	61.83	66.48		
POD6 WBC	Surgical Group	45	8.29	3.42	−1.458	0.149
	Conservative Group	35	9.52	4.14		
POD6 CRP	Surgical Group	45	10.52	12.96	−1.652	0.103
	Conservative Group	35	15.42	13.42		
POD6 PCT	Surgical Group	45	0.38	0.64	−0.892	0.375
	Conservative Group	35	0.52	0.72		
POD6 IL−6	Surgical Group	45	8.61	9.38	0.427	0.671
	Conservative Group	35	21.73	20.90		

p: Significance level of t-test between training set and validation set (*p* < 0.05).

**Table 3:** Comparison of postoperative recovery time between groups.

Parameters	Surgical group (*n* = 45)	Conservation group (*n* = 35)	Statistical measures	*P*
Hospital stay (days)	9.56 ± 2.82	9.29 ± 2.79	t = 0.44	0.663
Time to first flatus (hours)	14.00 ± 6.95	33.14 ± 20.94	U = 132.5	<0.001
Abdominal pain duration (hours)	34.24 ± 10.88	63.77 ± 28.78	U = 127.0	<0.001

### 3.4 Comparison of general characteristics between groups

#### 3.4.1 Postoperative hospital stay

No significant difference was found between the surgical treatment group (*n* = 45) and conservative treatment group (*n* = 35) (surgical group: 9.56 ± 2.82 days vs. conservative group: 9.29 ± 2.79 days; independent samples t-test: t = 0.44, *P* = 0.663, Cohen's d = 0.09).

#### 3.4.2 Postoperative gastrointestinal function recovery

The first flatus time in the surgical treatment group was significantly shorter than in the conservative treatment group (surgical group: 14.00 ± 6.95 h vs. conservative group: 33.14 ± 20.94 h; Mann–Whitney *U* test: U = 132.5, *P* < 0.001, effect size *r* = 0.72). The 95% confidence interval for the group difference was [−23.21, −14.06] hours, suggesting surgical intervention accelerates gastrointestinal function recovery.

#### 3.4.3 Duration of postoperative abdominal pain

The abdominal pain relief time in the surgical treatment group was significantly shorter than in the conservative treatment group (surgical group: 34.24 ± 10.88 h vs. conservative group: 63.77 ± 28.78 h; Mann–Whitney *U* test: U = 127.0, *P* < 0.001, effect size *r* = 0.75). The 95% confidence interval for the group difference was [−37.21, −21.83] hours, suggesting surgical intervention rapidly alleviates abdominal pain symptoms.

## 3 Discussion

To date, long-term clinical studies domestically and internationally have indicated that the occurrence of pediatric acute appendicitis is closely associated with factors such as fecalith obstruction, ischemic necrosis of appendiceal tissue, reactive hyperplasia of the appendiceal wall, and gastrointestinal dysfunction. However, the exact etiology remains unconfirmed (20). If pediatric appendicitis is not promptly and appropriately managed, it can easily progress to periappendiceal abscess (21). Currently, there remains controversy regarding optimal treatment approaches for pediatric periappendiceal abscess globally, with no definitive consensus (22, 23). This study systematically compared inflammatory markers and general recovery profiles in 80 children with periappendiceal abscess treated either conservatively or with laparoscopic surgery. The results demonstrated that laparoscopic surgery provided significant advantages in promoting postoperative recovery, while both groups showed comparable efficacy in improving inflammatory indicators. The following discussion elaborates on these findings in the context of current medical evidence.

### 3.1 Analysis of therapeutic effects differences

The advantages of laparoscopic surgery in general recovery may be attributed to its minimally invasive characteristics. This procedure rapidly eliminates the infection source by thoroughly clearing the pus cavity under direct vision, removing the diseased appendix, and effectively draining residual pus. Compared to the passive anti-infection model of conservative treatment that relies on antibiotics, surgical debridement aligns more closely with fundamental principles of surgical infection management (24). Additionally, intraoperative separation of adhesions around the appendix prevents poor drainage due to local encapsulation, thereby reducing risks of abscess recurrence and complications like chronic pelvic inflammation. Notably, the early recovery of immune function indicators in the surgical group suggests that intervention may accelerate overall recovery by alleviating immunosuppression and promoting active immune responses.

### 3.2 Analysis of changes in inflammatory indicators

Although both groups exhibited significant decreases in inflammatory markers (e.g., CRP, WBC) post-treatment, laparoscopic surgery showed no statistically superior advantage in these metrics. This phenomenon may relate to the following mechanisms: Conservative treatment effectively controls systemic inflammation through adequate antibiotics and supportive care, while surgical trauma itself acts as a stressor that transiently activates nonspecific immune responses, resulting in an early postoperative plateau of inflammatory indicators. However, dynamic monitoring data suggest that the long-term benefits of surgical debridement derive from more complete eradication of the infection focus rather than short-term stress reactions.

### 3.3 Evidence-based analysis of clinical decision-making

The findings suggest that laparoscopic surgery may serve as the preferred treatment for pediatric periappendiceal abscess when surgical indications are strictly met. Early surgical intervention for children exhibiting persistent high fever, progressive abscess enlargement, or intestinal obstruction can shorten disease duration and reduce long-term complication risks. However, excessive pursuit of surgical intervention may increase anesthesia- and surgery-related risks, particularly for young children or patients with severe systemic infections. Clinical practice thus requires comprehensive assessment of disease severity, surgical tolerance, and medical resource availability to formulate individualized treatment plans.

### 3.4 Study limitations and future research directions

As a single-center retrospective study, this research has inherent limitations. The small sample size may limit statistical power, especially for detecting rare complications. Although baseline characteristics were statistically matched, potential confounding factors cannot be fully excluded. Future multicenter prospective randomized controlled trials should incorporate additional clinical endpoints (e.g., quality of life, long-term recurrence rates) and utilize dynamic biomarker monitoring (e.g., IL-6/IL-10 ratio, sTREM-1) to elucidate surgical impacts on inflammatory regulation networks. Furthermore, artificial intelligence-based imaging systems warrant exploration for quantifying abscess extent and optimizing surgical timing.

### 3.5 Translational medical significance

This study provides new evidence-based foundations for standardizing the treatment of pediatric periappendiceal abscess. In the era of precision medicine, achieving dynamic equilibrium between minimally invasive surgical approaches and precision anti-infection conservative strategies represents a critical direction for future clinical research. With the widespread adoption of laparoscopic techniques and related instruments in pediatrics, alongside breakthroughs in molecular diagnostics for precision subtyping of infectious diseases, individualized clinical decision-making will become increasingly accurate and effective, ultimately maximizing therapeutic benefits for pediatric patients.

Based on the findings, this study preliminarily confirms that laparoscopic surgery for pediatric periappendiceal abscess can more effectively improve immune function, promote inflammation resolution, enhance therapeutic efficacy, and shorten treatment duration, providing more precise guidance for clinical decision-making. However, further validation of its long-term efficacy and safety through high-quality clinical research is required. Future studies should focus on establishing a multidimensional efficacy evaluation system and delve into the pathophysiological mechanisms of surgical intervention to provide higher-level evidence-based medical evidence for clinical practice.

## Data Availability

The original contributions presented in the study are included in the article/Supplementary Material, further inquiries can be directed to the corresponding author.

## References

[1] Nazarey PP Stylianos S Velis E Triana J Diana-Zerpa J Pasaron R. Treatment of suspected acute perforated appendicitis with antibiotics and interval appendectomy. J Pediatr Surg. (2014) 49:447–50. 10.1016/j.jpedsurg.2013.10.001 24650475

[2] Naqvi SA Thompson GC Joffe AR Blackwood J Martin D-A Brindle M. Cytokines and chemokines in pediatric appendicitis: a multiplex analysis of inflammatory protein mediators. Mediators Inflamm. (2019) 2019:1–13. 10.1155/2019/2359681 PMC6409077 30918467

[3] Brady Z Ramanauskas F Cain TM Johnston PN. Assessment of paediatric CT dose indicators for the purpose of optimisation. Br J Radiol. (2012) 85(1019):1488–98. 10.1259/bjr/28015185 22844033 PMC3500792

[4] Goldin AB Khanna P Thapa M McBroom JA Garrison MM Parisi MT. Revised ultrasound criteria for appendicitis in children improve diagnostic accuracy. Pediatr Radiol. (2011) 41:993–9. 10.1007/s00247-011-2018-2 21409546

[5] Deelder JD Richir MC Schoorl T Schreurs WH. How to treat an appendiceal inflammatory mass: operatively or nonoperatively? J Gastrointest Surg. (2014) 18:641–5. 10.1007/s11605-014-2460-1 24493295

[6] Bahram MA. Evaluation of early surgical management of complicated appendicitis by appendicular mass. Int J Surg. (2011) 9(1):101–3. 10.1016/j.ijsu.2010.10.006 20965290

[7] Kumar SS Collings AT Lamm R Haskins IN Scholz S Nepal P. SAGES Guideline for the diagnosis and treatment of appendicitis. Surg Endosc. (2024) 38(6):2974–94. 10.1007/s00464-024-10813-y 38740595

[8] Robert B Chivot C Rebibo L Sabbagh C Regimbeau J-M Yzet T. Percutaneous transgluteal drainage of pelvic abscesses in interventional radiology: a safe alternative to surgery. J Visc Surg. (2016) 153(1):3–7. 10.1016/j.jviscsurg.2015.10.006 26522506

[9] Tannoury J Abboud B. Treatment options of inflammatory appendiceal masses in adults. World J Gastroenterol. (2013) 19(25):3942–50. 10.3748/wjg.v19.i25.3942 23840138 PMC3703180

[10] Michelson KA Bachur RG Rangel SJ Finkelstein JA Monuteaux MC Goyal MK. Disparities in diagnostic timeliness and outcomes of pediatric appendicitis. JAMA Netw Open. (2024) 7(1):e2353667. 10.1001/jamanetworkopen.2023.53667 38270955 PMC10811560

[11] CODA Collaborative, Flum DR Davidson GH Monsell SE. A randomized trial comparing antibiotics with appendectomy for appendicitis. N Engl J Med. (2020) 383(20):1907–19. 10.1056/NEJMoa2014320 33017106

[12] Vaos G Dimopoulou A Gkioka E Zavras N. Immediate surgery or conservative treatment for complicated acute appendicitis in children? A meta-analysis. J Pediatr Surg. (2019) 54(7):1365–71. 10.1016/j.jpedsurg.2018.07.017 30115448

[13] Blakely ML Williams R Dassinger MS Eubanks JW Fischer P Huang EY. Early vs interval appendectomy for children with perforated appendicitis. Arch Surg. (2011) 146:660–5. 10.1001/archsurg.2011.6 21339413

[14] Guida E Pederiva F Grazia MD Codrich D Lembo MA Scarpa MG. Perforated appendix with abscess. Int J Surg Case Rep. (2015) 12:15–8. 10.1016/j.ijscr.2015.05.003 25985296 PMC4485681

[15] Myers AL Williams RF Giles K Waters TM Eubanks JW Hixson DS. Hospital cost analysis of a prospective, randomized trial of early vs interval appendectomy for perforated appendicitis in children. J Am Coll Surg. (2012) 214:427–35. 10.1016/j.jamcollsurg.2011.12.026 22342789

[16] Byun J Park S Hwang SM. Diagnostic algorithm based on machine learning to predict complicated appendicitis in children using CT, laboratory, and clinical features. Diagnostics. (2023) 13(5):923. 10.3390/diagnostics13050923 36900066 PMC10001049

[17] Kaya B Sana B Eris C Karabulut K Bat O Kutanis R. The diagnostic value of D-dimer, procalcitonin and CRP in acute appendicitis. Int J Med Sci. (2012) 9(10):909–15. 10.7150/ijms.4733 23236260 PMC3520016

[18] Menteş O Eryılmaz M Harlak A Oztürk E Tufan T. The value of serum fibrinogen level in the diagnosis of acute appendicitis. Turkish J Trauma Emerg Surg. (2012) 18(5):384–8. 10.5505/tjtes.2012.58855 23188598

[19] Ortiz-Ley JD Rodríguez-Zepeda ADR Paque-Bautista C González AP Cano-Rodríguez MT Cortés-Salim P. Índices inflamatorios predictores de apendicitis aguda complicada en niños [inflamatory indexes predictors of complicated acute apendicitis in children]. Rev Med Inst Mex Seguro Soc. (2023) 61(Suppl 2):S161–70. 38011620 PMC10769564

[20] Lin Z Zeng H Cai S Chen F Wang X Wu D. Effects of rhubarb peony decoction combined with antibiotics in treating pediatric periappendiceal abscess. Front Pediatr. (2023) 11:1112034. 10.3389/fped.2023.1112034 37063672 PMC10090493

[21] Bickell NA Aufses AH Rojas M Bodian C. How time affects the risk of rupture in appendicitis. J Am Coll Surg. (2006) 202:401–6. 10.1016/j.jamcollsurg.2005.11.016 16500243

[22] Feng SJ Zhou YF Yang JF Shen HZ Cui GX Zhang XF. Is appendoscope a new option for the treatment of acute appendicitis? World J Gastroenterol. (2024) 30(28):3386–92. 10.3748/wjg.v30.i28.3386 39091716 PMC11290393

[23] Zhang X Zheng X Jin H Li A Zhang H Zhang D. Efficacy and safety of different antibiotic treatment versus surgical treatment for acute appendicitis: a network meta-analysis. World J Surg. (2024) 48(12):2843–54. 10.1002/wjs.12382 39500855

[24] Tang HW Wang ZG Huang JH Zhang GQ Xu YF Zheng LL. The development and application of pediatric complicated appendicitis prediction model. Technol Health Care. (2023) 31(6):2319–29. 10.3233/THC-230285 37522234

